# Multiple Family Members With Delayed Cord Separtion and Combined Immunodeficiency With Novel Mutation in *IKBKB*

**DOI:** 10.3389/fped.2020.00009

**Published:** 2020-02-14

**Authors:** Zobaida Alsum, Mofareh S. AlZahrani, Hamoud Al-Mousa, Nouf Alkhamis, Abdulkareem A. Alsalemi, Hanan E. Shamseldin, Fowzan S. Alkuraya, Abdullah A. Alangari

**Affiliations:** ^1^Department of Pediatrics, King Saud University Medical City, Riyadh, Saudi Arabia; ^2^Royal Commission Medical Center, Yanbu, Saudi Arabia; ^3^Department of Pediatrics, King Faisal Specialist Hospital and Research Center, Riyadh, Saudi Arabia; ^4^Maternity and Children's Hospital, Ministry of Health, Najran, Saudi Arabia; ^5^Department of Genetics, King Faisal Specialist Hospital and Research Center, Riyadh, Saudi Arabia; ^6^Department of Anatomy and Cell Biology, College of Medicine, Alfaisal University, Riyadh, Saudi Arabia; ^7^Saudi Human Genome Program, King Abdulaziz City for Science and Technology, Riyadh, Saudi Arabia; ^8^Department of Pediatrics, College of Medicine, King Saud University, Riyadh, Saudi Arabia

**Keywords:** inhibitor of kappa kinase beta/inhibitor of kappa kinase 2, *IKBKB*, combined immunodeficiency, hematopoietic stem cell transplant, delayed separation of the umbilical cord

## Abstract

**Background:** Inhibitor of kappa kinase 2 (IKK2) deficiency is a recently described combined immunodeficiency. It undermines the nuclear factor-kappa B (NF-κB) activation pathway.

**Methods:** The clinical and immunological data of four patients diagnosed with combined immunodeficiency (CID) from two related Saudi families were collected. Autozygosity mapping of all available members and whole exome sequencing of the index case were performed to define the genetic etiology.

**Results:** The patients had early onset (2–4 months of age) severe infections caused by viruses, bacteria, mycobacteria, and fungi. They all had hypogammaglobulinemia and low absolute lymphocyte count. Their lymphocytes failed to respond to PHA mitogen stimulation. A novel homozygous non-sense mutation in the *IKBKB* gene, c.850C>T (p. Arg284*) was identified in the index patient and segregated with the disease in the rest of the family. He underwent hematopoietic stem cell transplantation (HSCT) from a fully matched sibling with no conditioning. The other three patients succumbed to their disease. Interestingly, all patients had delayed umbilical cord separation.

**Conclusion:** IKK2 deficiency causes CID with high mortality. Immune reconstitution with HSCT should be considered as early as possible. Delayed umbilical cord separation in CID patients may be a clue to IKK2 deficiency.

## Introduction

Nuclear factor-kappa B (NF-kB) is a transcription factor that plays a major role in various biological processes including the immune system development, activation, and regulation. This transcription factor complex is held inactive in the cytoplasm by the inhibitor of NF-kB (IκB). The classical activation pathway of NF-kB is triggered by a large number of extracellular stimuli through different receptors, which result in the phosphorylation of IκB by IκB kinase (IKK complex; IKKα, IKKβ/IKK2, and IKKγ/NEMO) and the subsequent release of NF-kB complex so it can translocate into the nucleus and drive target gene expression (1).

Different primary immunodeficiency disorders result from genetic defects that involve components linked to the NF-kB activation pathway (2). IKK2 deficiency is a recently described combined immunodeficiency disease that leads to impairment of NF-kB signaling (3). Affected patients develop early severe and recurrent infections caused by bacterial, viral, fungal, and mycobacterial organisms. Unlike patients with IKKα and IKKγ mutations, IKK2-deficient patients do not typically have ectodermal dysplasia. Indeed, out of 22 patients with IKK2 deficiency described in the literature, only one patient has ectodermal dysplasia in the form of conical teeth (3–7).

Herein, we describe the clinical manifestations and the immunologic features of four Saudi patients from two related families with a diagnosis of combined immunodeficiency due to novel homozygous non-sense mutation in *IKBKB*.

## Methods

The medical records of four Saudi patients from two related families with a diagnosis of combined immunodeficiency were reviewed. The clinical and immunological data were collected. Autozygosity mapping of all available members and whole exome sequencing of the index member were performed as previously described (8). Written consent was obtained from the parents. The study was approved by the Research Advisory Council at King Faisal Specialist Hospital and Research Center (RAC# 2121053).

## Results

**Figure 1:**
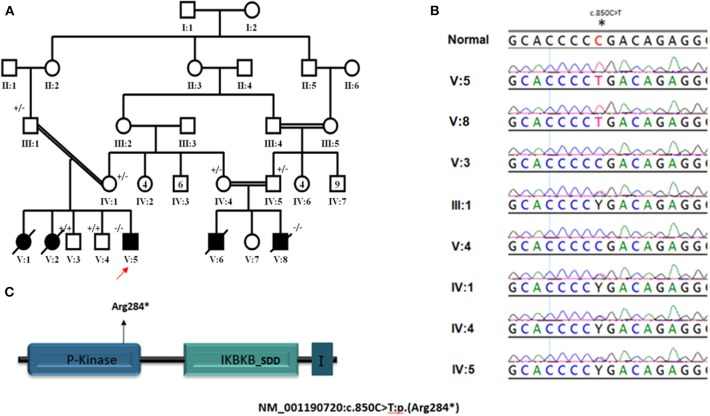
**(A)** Pedigree of study family. ± represent segregation status of different individuals. **(B)** Segregation of NM_001190720: c.850C>T mutation in parents and recruited unaffected siblings. **(C)** Cartoons for IKBKB transcript and protein domains; arrows point to the position of mutated base. I in last protein's domain stand for I-kappa-kinase-beta NEMO binding domain. *Indicates nonsense mutation resulting in termination of translation (truncated protein).

Our patients (V:1, V:2, V:5, and V:8) belong to two related Saudi families (Figure 1A). They had early onset (2–4 months of age) severe infections caused by viruses, bacteria, mycobacteria, and fungi. The organisms include *Klebsiella pneumonuia, Candida albicans*, CMV, and BCG (Table 1), which were associated with different clinical manifestations (Table 1). Interestingly, all four patients had delayed umbilical cord separation at 2 months. They all displayed hypogammaglobulinemia. Where data is available, they had low absolute lymphocyte count (420–2,680 cells/mm^3^), and their lymphocytes failed to respond to PHA mitogen (Table 1). A novel homozygous non-sense mutation in the *IKBKB* gene, c.850C>T (p. Arg284*) (Figures 1B,C) was identified in patient V:5 within the candidate autozygome, and Sanger sequencing confirmed its segregation with the disease in the remaining living siblings and parents (Figures 1A,B). Three patients (V:1, V:2, and V:8) succumbed to their disease's infectious complications before commencing hematopoietic stem cell transplantation (HSCT).

**Table 1:** The clinical and immunological characterization.

	**V:1**	**V:2**	**V:5**	**V:8**
Cord separation	2 months	2 months	2 months	2 months
Onset	4 months	3 months	3 months	2 months
Infections	Disseminated BCGitis	Perinatal CMV, *Klebsiella pneumoniae* sepsis, UTI, and pneumonia	Oral candidiasis and Disseminated BCGitis	*Klebsiella pneumoniae* sepsis and meningitis
Others	Extensive Maculopapular skin rash Pancytopenia Hepatosplenomegaly	Intracranial calcification Chorioretinitis Microcephaly Axial hypotonia Hepatosplenomegaly	Hepatosplenomegaly Maculopapular rash	
IgG^*^	0.75 g/L	1.39 g/L	0.574 g/L	2.679 g/L
IgM^*^	0.56 g/L	0.26 g/L	Undetectable	0.268 g/L
IgA^*^	Undetectable	Undetectable	Undetectable	0.425 g/L
IgE^*^	NA	Undetectable	Undetectable	NA
WBC	NA	NA	8,300/mm^3^	NA
ANC	NA	NA	5,890/mm^3^	NA
ALC^*^	420/mm^3^	2,680/mm^3^	1,030/mm^3^	NA
CD3^*^	260/mm^3^	NA	665/mm^3^ (65%)	NA
CD4^*^	210/mm^3^	NA	493/mm^3^ (48%)	NA
CD8^*^	50/mm^3^	NA	126/mm^3^ (12%)	NA
CD19^*^	50/mm^3^	NA	264/mm^3^ (26%)	NA
CD16/56^*^	100/mm^3^	NA	68/mm^3^ (7%)	NA
PHA^*^	RR%: 8%	RR%: 15%	RR%: 20%	NA
Outcome	Died at 6 months	Died at 8 months	Transplanted	Died at 3 months

* *Normal range for age: at 3 months: IgG (1.76–5.81 g/L), IgM (0.24–0.89 g/L), IgA (0.046–0.46 g/L), IgE (0.18–3.76 IU/ml). At 6 months: IgG (2.15–7.04 g/L), IgM (0.35–1.02 g/L), IgA (0.081–0.68 g/L), IgE (0.44–16.3 IU/ml). Absolute lymphocyte count (ALC): 4,054–7,048 cells/mm_3_, CD3: 3,100–4,800 cells/mm^3^ (61–70%), CD4: 2,200–3,300 cells/mm^3^ (38–51%), CD8: 1,100–1,700 cells/mm^3^ (18–27%), CD19: 1,100–1,900 cell/mm^3^ (20–27%), CD16/56: 300–700 cells/mm^3^ (5–11%), PHA (phytohemoagglutinin) RR%: 75–100%. RR, the ratio of the patient response to the mean of two normal control responses*.

Patient V:5 underwent HSCT from a fully matched sibling (10/10) at 19 months of age. To save his life and because of the disseminated BCGitis, he received no conditioning and no GVHD prophylaxis. The CD34 dose was 9.2 × 10^6^/kg. He had persistent lymphoid engraftment ranging from 7 to 40% (2 weeks to 2.5 years after HSCT) but no myeloid engraftment. He developed acute gut graft-vs.-host disease (GVHD) 2 months after HSCT that responded to cyclosporine A and steroid treatment. Six months later, he developed chronic gut GVHD that was controlled by the same medications. Although his lymphocyte subsets [CD3 2,409/mm^3^ (79%), CD4 1,211/mm^3^ (40%), CD8 1,183/mm^3^ (39%), CD19 296/mm^3^ (10%), CD16/56 307/mm^3^ (10%)] and their proliferation response to PHA (relative ratio 127%) showed good recovery, clinically, he did not show good immune reconstitution. He continued to suffer from disseminated BCGitis with bony involvement requiring frequent surgical interventions for faciozygomatic osteomyelitis. More than 4 years after the transplant, he was still on anti-mycobacterial medications (cycloserine, clarithromycin, ethambutol, and levofloxacin), monthly IVIG, and pentamidine prophylaxis. The last chimerism test was done at 4.5 years post-transplantation and showed 23% lymphoid engraftment and no myeloid engraftment. The severe disseminated BCGitis and history of GVHD precluded another trial of transplantation.

## Discussion

This is the largest cohort of IKK2 deficiency reported in the Saudi population. In general, the clinical features of our patients were not different from previously reported cases. Susceptibility to a wide range of infections caused by opportunistic and non-opportunistic organisms were commonality. The generalized maculopapular rash, hepatosplenomegaly, chorioretinitis, and CNS complications in our patients are probably related to the chronic CMV and disseminated BCG infections. All reported patients succumbed to their disease during infancy unless they had successful HSCT (Table 2). On average, the degree of lymphopenia was more profound in our patients (420–2,680/mm^3^) compared to others (1,520–11,850/mm^3^) (7). Interestingly, all our patients had delayed umbilical cord separation [normal separation occurs in 1–2 weeks (8)] Patient V:6, who died in infancy from a febrile illness before diagnosis and the identification of the genetic defect in the family, was also reported to have had delayed separation of the umbilical cord. None of the reported patients with IKK2 deficiency had delayed cord separation except one from Saudi Arabia, but with a different mutation (3–7). No neutrophil chemotaxis or adhesion assay was done for the patients. Delayed cord separation was reported in conditions that affect neutrophil number and/or compromise their function including leukocyte adhesion defects as well as MyD88 and IRAK4 deficiency, which are upstream to the NF-κB activation pathway (9–12). This feature may be a clue to IKK2 deficiency in patients with combined immunodeficiency. The mechanism by which *IKBKB* mutation may predispose to delayed cord separation is not clear. It has been reported that neutrophil infiltration was prominent during the cord separation process in healthy babies, and a defect in neutrophil chemotaxis and adhesion was illustrated in cases of leukocyte adhesion deficiency (13). Since all IKK2 deficiency patients in the literature did not exhibit delayed cord separation except one Saudi patient with a different mutation, it is likely that this feature is a result of a complex interaction between the *IKBKB* defect and other modifier genes.

**Table 2:** Summary of patients with IKK2 deficiency previously reported in the literature.

**Patient (references)**	**Onset**	**Clinical manifestation**	**HSCT**	**Age at 1st/2nd HSCT**	**Donor**	**HLA match**	**Conditioning**	**GVHD prophylaxis**	**Outcome/cause of death**	**Age at death**
P1 (7)	Unknown	Disseminated BCGitis	No	NA	NA	NA	NA	NA	Died/infection	<4 months
P2 (7)	3 months	Bacteremia, otitis media, pyelonephritis, pneumonia, tinea capitis, and oropharyngeal and perineal candidiasis. Causative organisms: *Klebsiella pneumoniae, E. coli* and polymicrobial gram negatives	No	NA	NA	NA	NA	NA	Died/infection	14 months
P3 (7)	2 weeks	Recurrent bacteremia, recurrent pneumonia, oral thrush, and disseminated HSV. Causative organisms are *E. coli*	Yes	19 months	Father	6/6	MAC	None	Died/not reported	7 years
P4 (7)	2.5 months	Disseminated BCGitis, disseminated CMV, disseminated adenovirus, bacteremia. Causative organisms: *Streptococcus pneumoniae, Stenotrophomonas maltophilia*	Yes	4 months	Half-sibling	10/10	None	None	Died/not reported	4 months
P5 (7)	1 month	Disseminated BCGitis, disseminated CMV, candidal meningitis, oral thrush, varicella zoster infection, and bacteremia. Causative organisms: *E. coli, Morganella morganni, Staphylococcus aureus*	No	NA	NA	NA	NA	NA	Died/infection	5 months
P6 (7)	3.5 months	Disseminated adenovirus, disseminated CMV, oral thrush, and bacteremia. Causative organisms: *E. coli, Pseudomonas aeroginosa*	Yes	4.5 months/not recorded	Mother/mother	10/10	None/MAC	Tacrolimus/methotrexate	Died/not reported	8 months
P7 (7)	2 months	*Listeria monocytogenes* bacteremia and meningitis and oral thrush	No	NA	NA	NA	NA	NA	Died/infection	2 months
P8 (7)	1 month	Oral thrush, pneumonia and bacteremia. Causative organism: *E. coli*	Yes	5 months	Unrelated	6/6	RIC	Tacrolimus/methotrexate	Died/not reported	2 years
P9 (7)	1 month	Oral and perineal candidiasis, RSV pneumonia, bacteremia and meningitis. Causative organisms: *Staphylococcus aureus, Enterococcus fecalis, Enterococcus faecium*, and *Leuconostoc lactic*	No	NA	NA	NA	NA	NA	Died/infection	15 months
P10 (7)	3 months	Oral thrush, bacteremia, and pneumonia. Causative organisms: Parainfluenza, *Mycoplasma pneumoniae, Staphylococcus hominis*, and *Enterococcus faecium*	Yes	7 months	Brother	10/10	MAC	Tacrolimus/methotrexate	Alive 7 years	NA
P11 (7)	2 months	Oral thrush, scabies, pneumonia, and meningitis. Causative organisms: rhinovirus and *Klebsiella pneumoniae*	No	NA	NA	NA	NA	NA	Died/infection	2.5 months
P12 (7)	3 months	Oral thrush, candidal UTI, bacteremia and brain abscesses, and pneumonia. Causative organisms: *Serratia marcescens, E. coli, Klebsiella pneumoniae*, RSV, coronavirus. Hemophagocytic lymphohistiocytosis	Yes	8 months	Unrelated	5/6	MAC	Tacrolimus/methotrexate	Alive 6 years	NA
P13 (7)	4 months	Sepsis	No	NA	NA	NA	NA	NA	Died/infection	2.5 months
P14 (7)	2.5 months	Disseminated BCGitis, candidemia, and bacteremia. Causative organisms: *E. coli, Stenotrophomonas maltophilia, Enterobacter cloacae*	No	NA	NA	NA	NA	NA	Died/infection	3.5 months
P15 (7)	Newborn (newborn screening)	None	Yes	2 months	Unrelated	5/6	MAC	Tacrolimus/methotrexate	Died	11 months
P16 (7)	Newborn (family history)	None	Yes	1 month	Unrelated	5/6	MAC	Tacrolimus/methotrexate	Alive 6 months	NA
P17 (5)	5 months	*Pneumocytis jirovecii pneumonia*, disseminated BCGitis	No	NA	NA	NA	NA	NA	Died/BCGitis and cardiac arrest	14 months
P18 (4)	2 months	Omphalitis, delayed separation of the umbilical cord, *Salmonella* sepsis, disseminated BCGitis, recurrent infections. Causative organisms: *Acinetobacter, Enterobacter, Stenotrophomonas*, and *Achromobacter* species, rotavirus, and *Candida*. Conical teeth.	No	NA	NA	NA	NA	NA	Died/massive bleeding after central line insertion	25 months
P19 (6)	5 months	*Candida*, BCGitis	Yes	Not recorded	Father	identical	MAC	Not recorded	Alive	
P20 (6)	11 months	*Candida*, BCGitis, rota virus	Yes	Not recorded	Unrelated Cord blood	Not recorded	MAC	Not recorded	Died/infection	2 months post HSCT
P21 (6)	7 months	*Candida*	Yes	Not recorded	Father	T-cell depleted haploidentical	MAC	Not recorded	Died/infection	1 year post HSCT
P22 (6)	6 months	*Candida, Klebsiella*, CMV	Yes	Not recorded	Mother	T-cell depleted haploidentical	MAC	Not recorded	Died/infection	13 months post HSCT

*MAC, myeloablative conditioning; RIC, reduced intensity conditioning; NA, not available*.

On the other hand, a gain-of-function (GOF) mutation in *IKBKB* leads to a relatively mild form of combined immunodeficiency, ectodermal dysplasia, and immune dysregulation, where affected patients may live to their fourth decade. Although both diseases result in hypogammaglobulinemia and lymphopenia, patients with *IKBKB* GOF mutation have lower number of naïve T lymphocytes with overactivated memory cells (14).

In our index patient, his unstable clinical condition and the disseminated BCGitis dictated the decision of non-conditioned HSCT. The fact that he had a sustained lymphoid engraftment without conditioning is likely due to the severe T-cell dysfunction in this type of immunodeficiency. We do not have a clear explanation why our index case did not clear the disseminated BCGitis inspite of the recovered *in vitro* lymphoproliferative response to PHA. One possibility is that the donor cells are anergic to BCG, but we could not test for that. So far, 13 out of 27 patients with IKK2 deficiency including our cohort had undergone HSCT. Only five were alive at the time of reporting, while those who did not receive HSCT succumbed to their disease in their infancy or early childhood. All survivors received myeloablative conditioning except our patient, while the remaining eight patients received non-myeloablative conditioning (*n* = 5), reduced intensity conditioning (*n* = 1), or no conditioning (*n* = 2) (14). Although it is difficult to draw a conclusion from such limited number of reported patients, it seems that no conditioning or reduced intensity conditioning are not sufficient to cure the disease, and myeloablative protocol is needed to establish a good immune reconstitution.

## Data Availability Statement

All datasets generated for this study are included in the article/supplementary material.

## Ethics Statement

The studies involving human participants were reviewed and approved by The Research Advisory Council (RAC) at King Faisal Specialist Hospital and Research Center (RAC# 2121053). Written informed consent to participate in this study was provided by the participants' legal guardian/next of kin.

## Author Contributions

ZA acquired data and wrote the manuscript. FA and HS designed and conducted the genetic study. HA-M contributed to writing the transplantation and post transplantation part of the manuscript. MA, NA, and AAls substantially contributed to the acquisition and analysis of the clinical data for the work. AAla acquired data, supervised, critically revised and edited the work for intellectual content. All authors provided approval for publication of the content and agree to be accountable for all aspects of the work in ensuring that questions related to the accuracy or integrity of any part of the work are appropriately investigated and resolved.

### Conflict of Interest

The authors declare that the research was conducted in the absence of any commercial or financial relationships that could be construed as a potential conflict of interest. The reviewer SP declared a past co-authorship with the author HA-M to the handling editor.
